# A transcriptome analysis of *Benincasa hispida* revealed the pathways and genes involved in response to *Phytophthora melonis* infection

**DOI:** 10.3389/fpls.2022.1106123

**Published:** 2022-12-22

**Authors:** Jinsen Cai, Songguang Yang, Wenrui Liu, Jinqiang Yan, Biao Jiang, Dasen Xie

**Affiliations:** ^1^ Guangdong Key Laboratory for New Technology Research of Vegetables, Vegetable Research Institute, Guangdong Academy of Agricultural Sciences, Guangzhou, China; ^2^ Guangdong Laboratory for Lingnan Modern Agriculture, Guangzhou, China

**Keywords:** RNA-Seq, *Benincasa hispida*, *Phytophthora melonis*, wilt disease, resistanceassociated gene

## Abstract

Wilt disease caused by *Phytophthora melonis* infection is one of the most serious threats to *Benincasa hispida* production. However, the mechanism of the response of *B. hispida* to a *P. melonis* infection remains largely unknown. In the present study, two *B. hispida* cultivars with different degrees of resistance to *P. melonis* were identified: B488 (a moderately resistant cultivar) and B214 (a moderately susceptible cultivar). RNA-seq was performed on *P. melonis*-infected B488 and B214 12 hours post infection (hpi). Compared with the control, 680 and 988 DEGs were respectively detected in B488 and B214. A KEGG pathway analysis combined with a cluster analysis revealed that phenylpropanoid biosynthesis, plant-pathogen interaction, the MAPK signaling pathway-plant, and plant hormone signal transduction were the most relevant pathways during the response of both B488 and B214 to *P. melonis* infection, as well as the differentially expressed genes in the two cultivars. In addition, a cluster analysis of transcription factor genes in DEGs identified four genes upregulated in B488 but not in B214 at 6 hpi and 12 hpi, which was confirmed by qRT-PCR. These were candidate genes for elucidating the mechanism of the *B. hispida* response to *P. melonis* infection and laying the foundation for the improvement of *B. hispida*.

## Introduction

In nature, interactions with microorganisms are unavoidable during the life-cycle of a plant. A large number of these microbes are plant pathogens; for example, pathogens in the genus *Phytophthora* (Chialva et al., 2022). *Phytophthora* belongs to the oomycete family, and some species, including *P. infestans*, *P. ramorum*, *P. sojae*, *P. nicotianae*, *P. capsica*, and *P. cinnamomi* are well-known pathogens that cause disease and significant losses in important agricultural and forestry crops worldwide. (Kamoun et al., 2015; Mora-Sala et al., 2022). Generally, *Phytophthora* species produce zoospores, which can reach speeds of up 250 mm/s when in swimming water (Appiah et al., 2005) before settling on the cell surface, germinating, and penetrating into the host cells. Under optimal conditions (i.e., 25-30°C and high relative humidity), a successful invasion is followed by growth and colonization of the host tissues, which eventually results in tissue collapse and sporulation, and this can turn into a serious epidemic within a few days or weeks (Lamour et al., 2012).

Pathogenic microbes destroy tissue and deprive host plants of nutrients, resulting in stunted growth and even death. Plants have developed defenses to survive pathogen attacks through long-term co-evolution with the pathogens (Zhou and Zhang, 2020; Jung et al., 2021). A plant has the capacity to recognize a diverse range of pathogen/microbe-associated molecular patterns (P/MAMP) *via* surface pattern-recognition receptors (PRRs), resulting in pattern-triggered immunity (PTI). Generally, PRRs are made up of receptor-like proteins and receptor kinases, which are found on the cell surface. Plants have also evolved resistance genes that encode nucleotide-binding leucine-rich repeat receptors, which recognize specific pathogen effectors, resulting in effector-triggered immunity (ETI) (Kourelis and van der Hoorn, 2018; Van de Weyer et al., 2019). The activation of immune receptors triggers downstream signaling, such as calcium influx, mitogen-activated protein kinase (MAPK) cascades, a burst of reactive oxygen species, accumulation of defense hormones, or the expression of immune marker genes (Tang et al., 2017; Tian et al., 2019; Huang et al., 2020). These effects can lead to defense execution processes such as callose deposition and the hypersensitive response (Jones and Dangl, 2006).

The activation of MAPK cascades is a major early signaling event in the plant defense response. There are two MAPK cascades; one is composed of MAP kinase kinase kinase (MEKK1), MAP kinase kinases (MKK1 and MKK2), and MAP kinase (MPK4) (Suarez-Rodriguez et al., 2007; Gao et al., 2008). A second cascade is composed of two MAPKKKs (MAPKKK3 and MAPKKK5), two MKKs (MKK4 and MKK5), and two MAPKs (MPK3 and MPK6) (Asai et al., 2002; Su et al., 2017). Additionally, positive feedback mechanisms are also involved, such as MAPKKK5, which is phosphorylated by MPK6 and further enhances the activation of MPK3/6, resulting in increased disease resistance (Bi et al., 2018). The receptors in the two layers (i.e., PTI and ETI) of the plant immune system detect MAMPs/effectors, which activate the MAPK cascades and trigger downstream signaling events such as plant hormone accumulation.

Plant hormones play a central role in plant immunity, and each different hormone regulates its own core pathway in the immune network. For instance, NPR1 and NPR3/NPR4, two classes of SA (salicylic acid) pathway receptors, interact with transcription factors (TGAs) to regulate transcriptional activation and repression (Zhang and Li, 2019). SA binds to receptors and induces defense gene expression by promoting NPR1 transcriptional activator activity and inhibiting NPR3/NPR4 transcriptional repression activity (Ding et al., 2018; Zhou and Zhang, 2020). The SA pathway is thought to be primarily directed at biotrophic pathogens (Zhang and Li, 2019). Additionally, JA (jasmonic acid) regulates another well-studied defense pathway (Wasternack and Song, 2017). The JA pathway can be subdivided into two branches: the ERF branch and the MYC branch. The ERF branch is co-regulated with ET (ethylene) and is associated with necrotrophic pathogens, while the MYC branch is co-regulated with ABA(abscisic acid) and provides general protection against chewing insects (Aerts et al., 2021).

In addition to signal molecules, secondary metabolites also play an important role in the plant defense response. The defensive functions of compounds produced by the phenylpropanoid pathways have been extensively researched. For example, lignification is caused by the deposition of lignin in the cell wall and serves as the first line of defense against pathogens (Bechinger et al., 1999; Denness et al., 2011). Lignin is one of the main components of the plant cell wall, acting as an anti-microbial compound by establishing a mechanical barrier, chemically modifying wall-degrading enzymes, or facilitating toxin diffusion, toxic precursor production, and free radical production. The genes involved in lignin production also act as signaling molecules to modulate the defense response (Naoumkina et al., 2010; Wang et al., 2015). Other compounds involved in the plant defense response include flavonoids, monolignols, phenolic acids, stilbenes, phytoalexins, and coumarins (Treutter, 2005; Zaynab et al., 2018). In addition, the phenylpropanoid pathway is associated with the SA signaling pathway. It has been discovered that PAL, a key enzyme in the phenylpropanoid pathway, participates in plant resistance by regulating SA levels (Zhang et al., 2017; Yuan et al., 2019).

*B. hispida* (wax gourd), the only member of the genus *Benincasa*, is widely cultivated in China and Southeast Asian countries; it is an important crop in the *Cucurbitaceae* family (Ma et al., 2021). One of the most serious threats to *B. hispida* production is wilt disease caused by a *Phytophthora* infection, with *P. melonis* as the primary pathogen (Ren et al., 2020). *P. melonis* falls into Clade 7b, as classified by genus-wide phylogenetic analyses of *Phytophthora* species (Blair et al., 2008). A *P. melonis* infection causes cucurbit blight; it bursts and spreads quickly during the rainy season, and roots, leaves, stems, and fruits are all susceptible. The underlying resistance mechanisms are still poorly understood, and transcriptome analysis is an effective method for understanding resistance mechanisms. According to the transcriptome analysis of the potato response to P. infestans infection, isolates with different virulence profiles can induce different defense responses at different time points (Duan et al., 2020); in the melon response to P. capsici infection, genes related to plant defense responses are stronger activated in a resistant cultivar than in a susceptible cultivar (Wang et al., 2020). In this study, high-throughput RNA-Seq was used to determine the transcriptome profiles of two *B. hispida* cultivars, B488 (a moderately resistant cultivar) and B214 (a moderately susceptible cultivar), that show significant differences in resistance to *P. melonis*. The identification of the differentially expressed genes in various signal pathways using GO (Gene Ontology) annotations, KEGG (Kyoto Encyclopedia of Genes and Genomes) enrichment, and cluster analysis led to the discovery of some genes that may be involved in *B. hispida* resistance to *P. melonis*.

## Materials and methods

### *B. hispida* growth, *P. melonis* cultivation

*B. hispida* cultivars B488 and B214 were provided by the Vegetable Research Institute, Guangdong Academy of Agricultural Sciences. *B. hispida* seedlings were cultivated at 28 °C in a greenhouse under a 16-h light/8-h dark photoperiod for 20 days until the two-leaf stage was reached. The *P. melonis* isolate FS-DY was cultivated on 10% V8 solid medium at 28 °C in the dark. The hyphae were transferred to 10% V8 liquid medium and cultivated 3 days for zoospore harvest.

### *P. melonis* infection

For RNA-seq: A *P. melonis* zoospore suspension (10^5^ zoospores per milliliter) was sprayed on the surface of the leaves of 20-day-old B488 and B214 seedlings, sterilized water was used as the control. The *P. melonis* inoculated seedlings and control seedlings were covered, separately. The treated seedlings were cultivated at 28 °C in the dark for 12 hours, then the leaves were dried by filter pepper before harvested in tinfoil and immediately snap-frozen in liquid nitrogen. Three biological replicates were performed for each treatment, and three seedlings for each replicate.

For resistance identification: Five milliliters of a *P. melonis* zoospore suspension (10^3^ zoospores per milliliter) was applied to each *B. hispida* seedling. The zoospore suspension was irrigated close to the stem; after irrigation, the seedlings were cultivated at 28 °C under a 16-h light/8-h dark photoperiod. The appearance of browning at the base of a seedling stem and lodging was recorded as death. The survival rate was calculated as the number of survival seedlings divided by the total number of seedlings. Survival rates were determined in triplicate for each cultivar. Ten seedlings were used in each experiment.

### RNA sequencing and differentially expressed genes analyses

RNA extraction and sequencing were performed by Shanghai Majorbio Bio-pharm Technology Co., Ltd, Genes Differential Expression and Gene Functional Enrichment Analyses were conducted on the online platform of the Majorbio Cloud Platform (www.majorbio.com). All the sequencing data were deposited in the NCBI Short Read Archive (SRA) database under the BioProject ID: PRJNA895846.

### Quantitative real-time RT-PCR

The Bio-Rad Real-time PCR system CFX96 and SYBR Premix (TranStart Green qPCR SuperMix, TransGen Biotech) were used to quantify gene expression. Total RNA extraction (EasyPure Plant RNA Kit, TransGen Biotech) and qRT-PCR were carried out according to the RNA extraction and SYBR Premix instructions, respectively. The specific primer pairs used are listed in **Supplementary Table 2**. Data were analyzed by the Livak method (Livak and Schmittgen, 2001) and expressed as a normalized relative expression level (2^-ΔΔCT^) of the respective genes, and the relative transcript level of each sample was normalized to actin (**Supplementary Table 2**). Six biological replicates were included in the experiment. The IBM SPSS version 21 software was used for statistical analysis, the *p* value was determined using Fisher’s protected LSD test.

## Results

### B488 and B214 had different resistance to *P. melonis*

**Figure 1:**
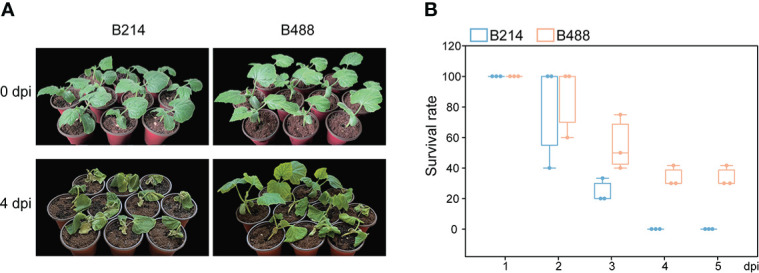
P*. melonis* resistance of B488 and B214. **(A)** Two-leaf stage B488 and B214 seedlings were irrigated with 5 ml of a *P. melonis* zoospore suspension (10^3^ zoospores per milliliter) for each seedling, pictures showed the state of B488 and B214 seedlings at 4 days post infection (dpi). **(B)** the survival rates of B488 and B214 were calculated daily for 5 days post infection.

To select varieties with different resistance to *P. melonis*, we assessed the resistance of 20 *B. hispida* cultivars based on field performance; two of these, B488 and B214, showed significant differences in resistance. The resistance of the two cultivars was reconfirmed using the root-irrigation method. Different concentrations of pathogen could affect the results and plants were hardly survival from a high concentration of pathogen. According to the experiment, we chased a moderate concentration (10^3^ zoospores per milliliter) to perform the root-irrigation experiment and the results showed that B488 is more resistant to *P. melonis* than B214 (**Figure 1A**), with survival rates of 55 ± 18.0% at 3 dpi, 33.8 ± 6.7% at 4 dpi and 33.8 ± 6.7% at 5 dpi for B448, while B214 had survival rates of 24.4 ± 7.7% at 3 dpi, 0 at 4 dpi and 0 at 5 dpi (**Figure 1B**). Taken together, these findings suggest that B214 and B488 are respectively a moderately susceptible and resistant cultivar to *P. melonis*.

### RNA-seq and quality evaluation

To understand the mechanism causing the differences in the resistance of B488 and B214 to *P. melonis* and the different expression patterns of genes in the early phase of B488 and B214 response to *P. melonis* infection, we performed the inoculation by spraying *P. melonis* zoospore suspension on the surface of the leaves of 20-day-old B488 and B214 seedlings. To ensure the success of infection, a high concentration (10^5^ zoospores per milliliter) of *P. melonis* zoospore were chased to perform the experiment. The differentially expressed genes (DEGs) were investigated by RNA sequencing (RNA-seq) 12 h after B488 and B214 were infected with *P. melonis* (PmI). Shanghai Majorbio Bio-pharm Technology Co., Ltd performed the RNA-seq procedure, and the data were analyzed on the Majorbio Cloud Platform online platform (www.majorbio.com).

Pearson correlation and principal component analyses (PCA) (**Supplementary Figure 1**) were used to compare the samples. The PCA1 had 37% variance and the PCA2 had 18.8% variance, each group was dispersed, and each group sample was gathered. **Supplementary Table 1** shows the data from the sequencing of all the samples. The proportion of bases with a quality of no less than 20 after filtration (Q20) was 97-98%, the proportion of bases with a quality of no less than 30 after filtration (Q30) was 92–94%, and the overall data sequencing error rate was less than 0.03%. The base numbers of G and C were 46–47% of the total base number. Mapping rates were greater than 96%. The above results revealed that the RNA-Seq data of these 12 samples were reliable and could be used for subsequent analysis.

### DEGs obtained and validation of DEGs by qRT-PCR

**Figure 2:**
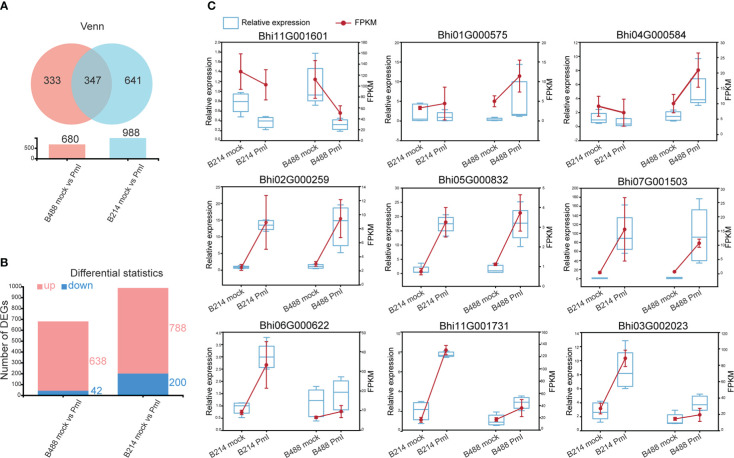
DEGs and the validation of selected DEGs using qRT-PCR. **(A)** Venn diagrammatic analysis of DEGs in B488 and B214. **(B)** up- and downregulated DEGs in B488 (mock infection vs. PmI) and B214 (mock infection vs. PmI). **(C)** Randomly selected DEGs expression tendency of qRT-PCR data compared with RNA-seq FKPM data.

The DEGs were identified based on the abundance of RNA-Seq reads and normalized to fragments per kilobase length per million reads (FPKM). A total of 680 (i.e., 638 upregulated and 42 downregulated) and 988 (i.e., 788 upregulated and 200 downregulated) DEGs that showed at least a twofold change in gene expression (q-values < 0.05) were respectively identified in B488 and B214 infected with *P. melonis* infection. A Venn diagram analysis revealed 347 common genes in the DEGs of B488 and B214, as well as 333, 641 distinct genes (**Figures 2A, B**).

To validate the RNA-Seq results, a quantitative real-time PCR (qRT-PCR) analysis was performed to detect the expression level of nine randomly selected DEGs for B488 and B214. Three genes were randomly selected from the common genes in DEGs of B488 and B214 (*Bhi02G000259*, *Bhi05G000832*, *Bhi07G001503*), three genes from the B488 DEGs of (*Bhi11G001601*, *Bhi01G000575*, *Bhi04G000584*), and three genes from the B214 DEGs of (*Bhi06G000622*, *Bhi11G001731*, *Bhi03G002023*). The qRT-PCR and FPKM data for the nine genes of B488 and B214 showed a similar variation tendency between a mock infection and the PmI (**Figure 2C**). These findings suggest that the transcriptome data are reliable.

### KEGG annotation analysis and GO annotations analysis

**Figure 3:**
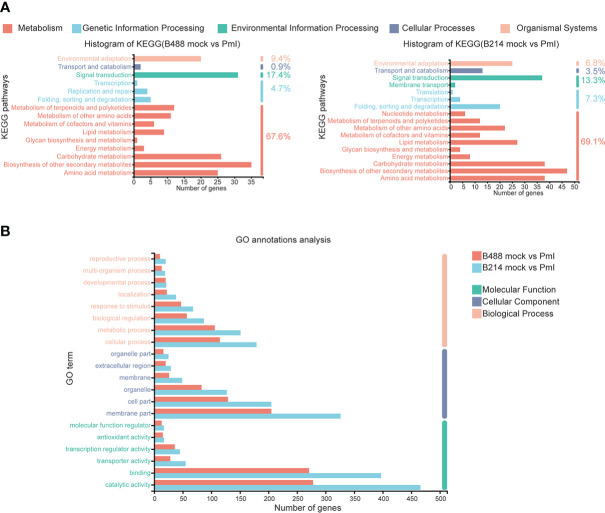
KEGG annotations analysis and GO annotations analysis of DEGs. **(A)** KEGG annotations analysis of the DEGs in B488 (mock infection vs. PmI) and B214 (mock infection vs. PmI), the number indicated the percent of gene enrichment in the pathway. **(B)** GO annotations analysis of DEGs in B488 (mock vs. PmI) and B214 (mock vs. PmI).

An annotation analysis of the B488 and B214 DEGs with KEGG shows that the majority of DEGs are involved in Metabolism, with up to 67.6% and 69.1% for B488 and B214, respectively. Carbohydrate metabolism, biosynthesis of other secondary metabolism, and amino acid metabolism were the pathways with the most enriched genes in the Metabolism category; folding, sorting and degradation, signal transport, transport and catabolism, and environmental adaptation were the pathways with the most enriched genes in the genetic information processing, environmental information processing, cellular processes and organismal systems categories, respectively (**Figure 3A**).

An analysis of GO annotations revealed that the most relevant terms in the process of *B. hispida* resistance to *P. melonis* were metabolic process and cellular processes in biological process, membrane part and cell part in cellular component, and catalytic activity and binding in molecular function. Furthermore, B214 had more enriched genes than B488 in almost all categories (**Figure 3B**). The KEGG pathways and GO terms mentioned above may be important in the *B. hispida* response to a *P. melonis* infection.

### KEGG enrichment analysis

**Figure 4:**
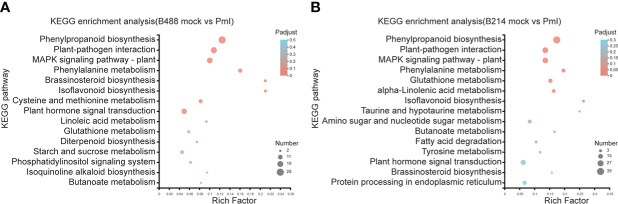
KEGG enrichment analysis of DEGs. **(A)** KEGG enrichment analysis of the DEGs in B488. **(B)** KEGG enrichment analysis of the DEGs in B214. The y-axis indicates the pathway names, and the x-axis indicates the rich factor. Rich factor refers to the ratio of the number of DEGs located in the KEGG pathway and the total number of genes in the KEGG pathway. The larger the rich factor the greater the degree of enrichment. The size of the dots indicates the number of genes in the pathway, while the color of the dots indicates the adjust *p*-value ranges.

*Phytophthora* was defined as a hemibiotrophic pathogen, and the first 18 hours were Biotrophy. In this early phase, hyphae pushed the host cell membrane inwards, forming a direct host-pathogen interface, and the plant cells were not destroyed. This is a critical stage of *Phytophthora* defense. The KEGG enrichment analysis was used to preserve the DEGs involved in this early phase of the defense response. The top four enrichment pathways for the moderately resistant cultivar B488 DEGs were phenylpropanoid biosynthesis (28 genes), plant-pathogen interaction (20 genes), MAPK signaling pathway (17 genes), and plant hormone signal transduction (16 genes). The top four enrichment pathways for the moderately susceptible cultivar B214 DEGs were phenylpropanoid biosynthesis (39 genes), plant-pathogen interaction (25 genes), MAPK signaling pathway (23 genes), and plant hormone signal transduction (20 genes) (**Figure 4**).

### DEGs involved in phenylpropanoid biosynthesis pathway

According to the KEGG enrichment analysis, the DEGs involved in the early phase (i.e., 12 hpi) of the *B. hispida* (i.e., B488 and B214) response to a *P. melonis* infection were enriched in the same primary pathways (i.e., Phenylpropanoid biosynthesis, Plant-pathogen interaction, MAPK signaling pathway – plant, and Plant hormone signal transduction). The DEGs were used to perform a cluster analysis to detect the expression profile of the genes in B488 and B214 that were enriched in the aforementioned pathways.

**Figure 5:**
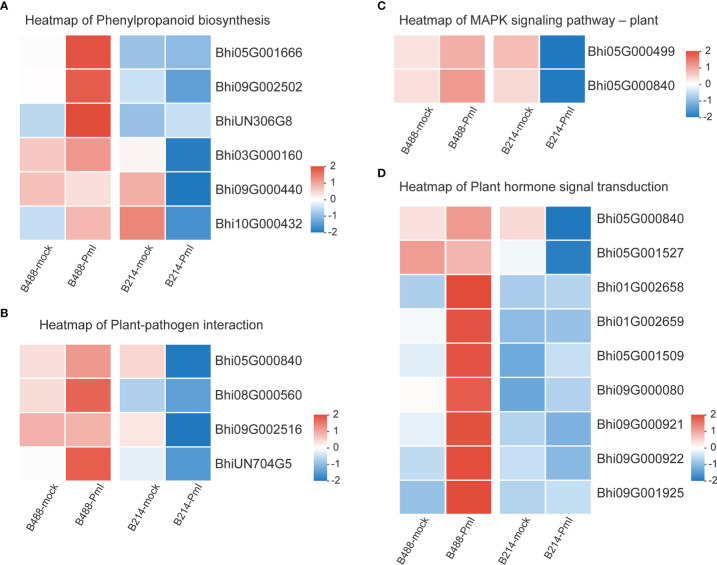
Heatmap of DEGs. **(A)** Heatmap of the DEGs in the phenylpropanoid biosynthesis pathway. **(B)** Heatmap of the DEGs in the plant pathogen interaction pathway. **(C)** Heatmap of the DEGs in the MAPK signaling-plant pathway. **(D)** Heatmap of the DEGs in the plant hormone signal transduction pathway. Log_10_(FPKM+1) values were used in the heatmap, and the average values of FPKM from the three biological replicates were used for log_10_ (FPKM+1) calculation. Red indicates high expression, and blue indicates low expression.

Genes involved in the Phenylpropanoid biosynthesis pathway were clustered into six subclusters based on the expression patterns in the cluster analysis (**Supplementary Figure 3**). In B488, but not in B214, the *P. melonis* infection induced three genes (Subcluster 4), which coded for peroxidase. In B214, but not in B488, the expression of an oxidoreductase (*Bhi03G000160*) and a transferase gene (*Bhi09G000440*) was reduced (subcluster 5). The phenylalanine ammonia lyase gene (*Bhi10G000432*) was downregulated in B214 but upregulated in B488 (subcluster 6) (**Figure 5A**; **Table 1**).

**Table 1:** Genes in subcluster 4, 5 and 6 of phenylpropanoid biosynthesis pathway.

subcluster	Gene id	NR description
4	*Bhi05G001666*	peroxidase P7-like
	*Bhi09G002502*	peroxidase 18
	*BhiUN306G8*	lignin-forming anionic peroxidase-like
5	*Bhi03G000160*	flavin-dependent oxidoreductase FOX2-like
	*Bhi09G000440*	quinate hydroxycinnamoyl transferase
6	*Bhi10G000432*	phenylalanine ammonia lyase-like

NR (Non-Redundant Protein Sequence Database).

### DEGs involved in the plant-pathogen interaction pathway

Plant-pathogen interaction is a pathway that consists of pathogen association receptors, signal transfer molecules, and resistant genes, and is the most important pathway for plant defense. The genes involved in the Plant-pathogen interaction pathway were clustered into six subclusters based on their expression patterns in the cluster analysis. Most genes in this pathway were induced by the *P. melonis* infection, with the exception of four genes in subcluster 5 that were upregulated in B488 but downregulated in B214 (**Supplementary Figure 4**; **Figure 5B**). These four genes were predicted to code for two 3-ketoacyl-CoA synthases, a protein kinase, and a basic form of pathogenesis-related protein 1 (**Table 2**).

**Table 2:** Genes in subcluster 5 of plant pathogen interaction pathway.

subcluster	Gene ID	NR description
5	*Bhi08G000560*	3-ketoacyl-CoA synthase 12-like
	*BhiUN704G5*	calcium-dependent protein kinase 29
	*Bhi05G000840*	basic form of pathogenesis-related protein 1-like
	*Bhi09G002516*	3-ketoacyl-CoA synthase 10

NR (Non-Redundant Protein Sequence Database).

### DEGs involved in the MAPK signaling pathway–plant

The MAPK cascade is an effective signaling transduction system that plays an important role in plant defense. In the cluster analysis of genes involved in the MAPK signaling pathway-plant (**Supplementary Figure 5**), most genes were induced by *P. melonis* infection, except for the genes in subcluster 6 (**Figure 5C**; **Table 3**). *Bhi05G000499* has been predicted to code for an LRR receptor kinase and may be upstream of the MAPK cascades. Reduction of the expression of *Bhi05G000499* in B214 but not in B488 during a P. melonis infection is an indication that a specific MAPK signaling pathway was more highly activated in B488 than in B214.

**Table 3:** Genes in subcluster 6 of MAPK signaling pathway–plant.

subcluster	Gene ID	NR description
6	*Bhi05G000840*	basic form of pathogenesis-related protein 1-like
	*Bhi05G000499*	LRR receptor-like serine/threonine-protein kinase ERL1

NR (Non-Redundant Protein Sequence Database).

### DEGs involved in plant hormone signal transduction pathway

To identify the hormones that participate in *B. hispida* response to *P. melonis* infection, a cluster analysis of genes involved in Plant hormone signal transduction was performed (**Supplementary Figure 6**). The results show that genes in subcluster 4 were downregulated by a *P. melonis* infection of B214 but not B488, and genes in subcluster 6 were upregulated in B488 but not in B214 (**Figure 5D**). With the exception of the *Bhi05G000840* gene, which was predicted to code for a basic form of pathogenesis-related protein 1-like and was related to the SA (salicylic acid) signaling pathway, the others were all involved in the auxin signaling pathway (**Table 4**), and the gene expression tendency led to a similar result; i.e., more auxin-induced/responsive protein was accumulated in B488 than B214 at 12 hpi, which suggests that auxin signaling is a key signaling pathway that contributes to the difference in resistance between B488 and B214 to a *P. melonis* infection.

**Table 4:** Genes in subcluster 4 and 6 of plant hormone signal transduction pathway.

subcluster	Gene id	NR description
4	*Bhi05G000840*	basic form of pathogenesis-related protein 1-like
	*Bhi05G001527*	auxin-responsive protein SAUR50-like
6	*Bhi01G002658*	auxin-induced protein 22A-like
	*Bhi01G002659*	auxin-induced protein AUX22-like
	*Bhi05G001509*	auxin-responsive protein
	*Bhi09G000080*	auxin-induced protein AUX22-like
	*Bhi09G000921*	auxin-induced protein 22D-like
	*Bhi09G000922*	auxin-induced protein AUX28-like
	*Bhi09G001925*	auxin-responsive protein IAA29-like

NR (Non-Redundant Protein Sequence Database).

### Transcription factor genes Involved in *B. hispida* response to *P. melonis* infection

**Figure 6:**
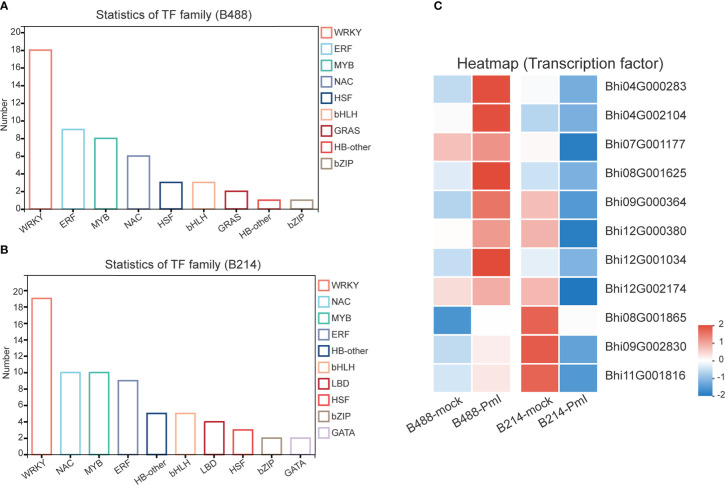
TF family of DEGs. **(A)** Statistics for TF in B488 (mock infection vs. PmI). **(B)** Statistics of TF in B214 (mock infection vs. PmI). **(C)** Heatmap of differentially expressed TF genes between B488 (mock infection vs. PmI) and B214 (mock infection vs. PmI).

It has been suggested that transcription factors (TFs) can play either a positive or negative role in the plant defense response. A total of 51 and 78 genes, respectively, of the DEGs of B488 and B214 are predicted to encode TF proteins. A total of 51 TF genes of the DEGs of B488 were categorized into 9 TF families according to statistics on the TF families (**Figure 6A**); the top three were the WRKY, ERF, and MYB families with 18, 9, and 8 members, respectively. The 78 TF genes in the B214 DEGs were classified into 19 TF families (**Figure 6B**), with the primary TF families of WRKY (19 genes), NAC (10 genes), and MYB (10 genes). A cluster analysis of TF genes involved in the B. hispida response to a P. melonis infection revealed that genes in subclusters 5 and 6 have different expression patterns between B488 and B214, with increased expression in B488 and decreased expression in B214 (**Supplementary Figure 7**; **Figure 6C**) (**Table 5**).

**Table 5:** Genes in subcluster 5 and 6 of TF genes.

subcluster	Gene id	NR description
5	*Bhi04G000283*	transcription factor MYB44-like
	*Bhi04G002104*	ethylene-responsive transcription factor ERF020
	*Bhi07G001177*	transcription factor bHLH49 isoform X3
	*Bhi08G001625*	NAC domain-containing protein 86
	*Bhi09G000364*	GATA transcription factor 9-like
	*Bhi12G000380*	axial regulator YABBY 5-like
	*Bhi12G001034*	NAC domain-containing protein 45
	*Bhi12G002174*	squamosa promoter-binding-like protein 16
6	*Bhi08G001865*	ethylene-responsive transcription factor 11
	*Bhi09G002830*	ethylene-responsive transcription factor 4-like
	*Bhi11G001816*	floral homeotic protein APETALA 2-like

NR (Non-Redundant Protein Sequence Database).

**Figure 7:**
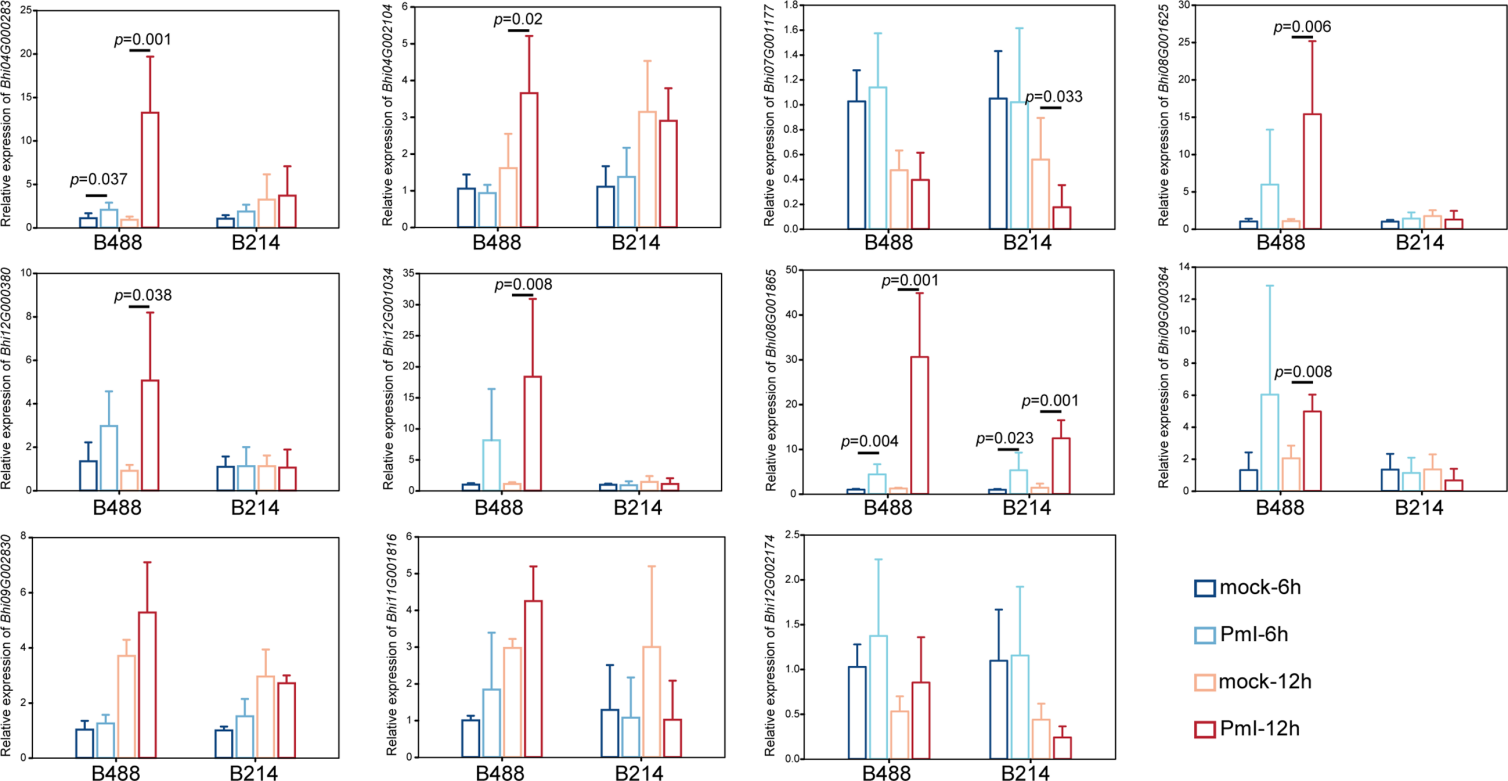
Expression pattern of TF genes. A *P. melonis* zoospore suspension (10^5^ zoospores per milliliter) was sprayed on surface of B488 and B214 leaves, sterilized water was sprayed on surface of B488 and B214 leaves as the control; leaves were harvested at 6 h and 12h for RNA extraction and subsequent qRT-PCR analysis, six biological replicates were performed, *p* value was determined using Fisher’s protected LSD test.

Detection of gene expression at 6 hpi and 12 hpi by qRT-PCR, allowed validation of the expression tendency of TF genes in B488 and B214 and identification of the TF genes induced by a *P. melonis* infection in the early phase. Six genes were significantly upregulated in B488 but were not significantly different in B214 (**Figure 7**), of which four (i.e., *Bhi04G000283*, *Bhi08G001625*, *Bhi09G000364* and *Bhi12G001034*) were upregulated at 6 hpi and 12 hpi and were predicted to code for MYB transcription factor, NAC transcription factor, GATA transcription factor and NAC transcription factor, respectively (**Table 5**). These four genes could be candidate genes that contribute to the different levels of resistance to *P. melonis* of B488 and B214.

## Discussion

*Benincasa hispida* is an important crop of the *Cucurbitaceae* family, and currently available cultivars are particularly vulnerable to *Phytophthora melonis*, one of the principal threats to wax gourd production. In this study, high throughput sequencing of two *B hispida* cultivars, B488 (a moderately resistant cultivar) and B214 (a moderately susceptible cultivar) with different degrees of resistance to *P melonis*, was performed to elucidate the mechanism of *B hispida* resistance to *P melonis* and identify the genes involved in the defense response. In comparison to a mock infection, 680 and 988 DEGs were respectively found in the presence of a *P. melonis* infection with B488 and B214 (**Figure 2A**). Phenylpropanoid biosynthesis, Plant-pathogen interaction, MAPK signaling pathway–plant, and Plant hormone signal transduction were discovered as the most active pathways when *B hispida* is attacked by *P melonis*. Comparing the expression patterns of the DEGs involved in the aforementioned pathways in B488 and B214 suggests some candidate genes that could contribute to the differences in the resistance of B488 and B214.

Phenylpropanoids are secondary metabolites that plants produce to improve their development and cope with biotic and abiotic stresses (Tohge et al., 2017). According to a KEGG enrichment analysis of DEGs at an early stage of a *P. melonis* infection of *B. hispida*, we discovered that phenylpropanoid biosynthesis is the most frequently seen enrichment pathway, and almost all the DEGs in this pathway are upregulated (**Figure S3**). One gene (*Bhi10G000432*), which was predicted to code phenylalanine ammonia lyase (PAL), was consistently upregulated in B488 and downregulated in B214 (**Figure 5A**). PAL is the gateway enzyme of the general phenylpropanoid pathway (Zhang and Liu, 2015); it has direct control over downstream metabolites such as lignin and flavonoid biosynthesis, which are linked to plant resistance. PAL can also contribute to plant resistance by regulating the SA levels (Zhang et al., 2017; Yuan et al., 2019). Direct evidence suggests that the overexpression of PAL will enhance the resistance and knockdown or silencing of PAL will increase the susceptibility of the plant to pathogens (Yadav et al., 2020). The higher expression of PAL in B488 than in B214 is associated with the higher resistance of the former, and the mechanism requires further investigation.

MAPK cascades are involved in a variety of defense events, including defense gene expression, phytoalexin and defense hormone biosynthesis, and stomatal immunity (Liu and Zhang, 2004; Li et al., 2012; Su et al., 2017). MAPKKKs are phosphorylated directly by PRRs or by receptor-like cytoplasmic kinases (RLCKs) that act downstream of PRRs in MAPK cascades, and evidence suggests that the same MAPKKK acting downstream of different PRRs may result in a different MAPK cascade activation (Yamada et al., 2016). Our study indicated a gene (*Bhi05G000499*) predicted to code for an LRR receptor kinase that is induced by *P. melonis* infection in B488 (**Figure 5C**). It is reasonable to hypothesize that this LRR receptor kinase activates a MAPK cascade in B488 that differs from the MAPK cascades activated in B214, resulting in the greater expression of genes involved in the Plant-pathogen interaction pathway (i.e., *Bhi08G000560*, *BhiUN704G5*, *Bhi05G000840*, *Bhi09G002516*) (**Figure 5B**).

Auxin, an important phytohormone, is involved in plant growth, development, and environmental stimuli, as well as plant-microbe interactions (Benjamins and Scheres, 2008). It is thought to increase susceptibility to the establishment of mutualistic or pathogenic microbes in plant tissues in plant-microbe interactions (Kunkel and Harper, 2018; McClerklin et al., 2018). In a study of soybean response to *Phytophthora sojae*, levels of auxin and related metabolites were significantly increased in soybean at 48 and 72 hpi, and increased auxin levels have been proposed to improve soybean susceptibility (Stasko et al., 2020). In this study, eight genes (i.e., *Bhi05G001527*, *Bhi01G002658*, *Bhi01G002659*, *Bhi05G001509*, *Bhi09G000080*, *Bhi09G000921*, *Bhi09G000922*, *Bhi09G001925*), predicted to code the AUX/IAA protein show different expression patterns in B488 and B214 after infection with *P melonis*, and it is believed that the Aux/IAA proteins are transcriptional co-regulators that function as repressors of the early auxin response genes at low auxin concentrations (Weijers and Wagner, 2016). In our study, the expressions of *Bhi01G002658*, *Bhi01G002659*, higher in the moderately resistant cultivar (B488), but not in the moderately susceptible cultivar (B214), reveal a stronger auxin signaling repression in B488 than in B214 during the early phase of *P melonis* infection. Furthermore, two indole-3-acetic acid-amido synthetase genes (i.e., *Bhi03G000746* and *Bhi09G001660*) were upregulated in B214 but not in B488 (**Figure S6**), implying that B214 accumulates more auxin than B488. Collectively, the auxin signaling repressor and auxin concentration are critical in the of the resistance *B hispida* to *P melonis*.

Many genes are reprogrammed during the defense response process, which is dependent on transcription factor regulation. Most TFs bind to a specific DNA sequence in the promoter region, and either activate or repress gene expression (Weirauch et al., 2014). A strategy for identifying TFs that function in plant defense is to first identify TF genes that show altered transcription levels during the initial period of the defense response. In this study, we found four TF genes are upregulated at 6 h and 12 h in B488 but not in B214 after infection with *P melonis*. *Bhi04G000283* encodes a MYB (myeloblastosis related) transcription factor, while *Bhi08G001625*, *Bhi12G001034*, and *Bhi09G000364* encode NAC [no apical meristem (NAM), Arabidopsis transcription activation factor (ATAF1/2), and cup-shaped cotyledon (CUC2)] transcription factor, NAC transcription factor and GATA transcription factor, respectively. MYB and NAC are two major TF families involved in plant defense (Ng et al., 2018). Indeed, MYB transcription factors participate in the defense response by modulating the biosynthesis of secondary metabolites (Wang et al., 2021). For instance, MYB34/51/122 contribute to resistance toward *Plectosphaerella cucumerina* exclusively through indolic glucosinolate biosynthesis (Frerigmann et al., 2016). NAC transcription factor positively regulates disease resistance by suppressing the ABA signaling pathway (Liu et al., 2018).GATA factors are evolutionarily conserved transcription factors that are found in animals, fungi, and plants. Previous studies of the biological roles of plant GATAs revealed that their major functions are associated with plant development (Schwechheimer et al., 2022), Nevertheless, we found that *Bhi09G000364* may be involved in the resistance to *P melonis* in *B hispida*, which is a new biological role for GATA factors

This study is the first report of the identification of genes that contribute to the defense of *B hispida* to *P melonis*. A COG (Clusters of Orthologous Groups) classification of DEGs in B488 and B214 shows that 59% and 55% of the genes, respectively, have unknown functions (**Supplementary Figure 2**), suggesting a great potential for the identification of *B hispida* resistance-associated genes. A subsequent functional analysis of these candidate genes could reveal the mechanism of the defense of *B hispida* against *P melonis* and identify molecular markers for developing *B hispida* cultivars with high levels resistance to *P melonis*.

## Data availability statement

The data presented in the study are deposited in the NCBI repository, accession number: PRJNA895846.

## Author contributions

JC, SY, and DX conceived and designed the experiments. JC and JY performed the experiments. JC, WL, and DX analyzed the data. JC wrote the manuscript. SY, WL, JY, BJ, and DX reviewed and revised the manuscript. All authors have read and approved the final version of the manuscript.
