# Supplementary material for: A transcriptome analysis of Benincasa hispida revealed the pathways and genes involved in response to Phytophthora melonis infection
Source: Front Plant Sci. 2022 Dec 22;13:1106123. doi: 10.3389/fpls.2022.1106123 (PMC9815465; doi:10.3389/fpls.2022.1106123)
Supplement: Supplementary file 1 [file DataSheet_1.docx]

**Supplementary Table 1**. Summary of the sequencing data.

| **Sample** | **Clean reads** | **Clean**  **bases** | **Error rate (%)** | **Q20 (%)** | **Q30 (%)** | **GC content (%)** | **Mapped reads (%)** |
| --- | --- | --- | --- | --- | --- | --- | --- |
| B488-PmI-3 | 49939276 | 7434369652 | 0.0264 | 97.51 | 92.76 | 46.55 | 48090966(96.3) |
| B488-PmI-2 | 53021704 | 7865918830 | 0.0257 | 97.78 | 93.41 | 46.77 | 51126621(96.43) |
| B488-PmI-1 | 51262340 | 7598392689 | 0.0252 | 97.97 | 93.88 | 46.69 | 49537233(96.63) |
| B488-mock-3 | 51652458 | 7652117624 | 0.0255 | 97.87 | 93.64 | 46.85 | 49790875(96.4) |
| B488-mock-2 | 58681680 | 8679929679 | 0.0257 | 97.8 | 93.45 | 46.68 | 56637256(96.52) |
| B488-mock-1 | 53398780 | 7924835332 | 0.026 | 97.66 | 93.13 | 46.62 | 51418962(96.29) |
| B214-PmI-3 | 48753418 | 7269217908 | 0.0262 | 97.61 | 92.98 | 46.02 | 47131517(96.67) |
| B214-PmI-2 | 54030524 | 8014613193 | 0.0257 | 97.79 | 93.43 | 46.11 | 52207402(96.63) |
| B214-PmI-1 | 49929632 | 7463574784 | 0.0263 | 97.55 | 92.81 | 46.66 | 48061947(96.26) |
| B214-mock-3 | 50239170 | 7473200287 | 0.0257 | 97.79 | 93.37 | 46.62 | 48436841(96.41) |
| B214-mock-2 | 50710476 | 7557515759 | 0.0259 | 97.7 | 93.2 | 46.66 | 48841429(96.31) |
| B214-mock-1 | 52507782 | 7818782651 | 0.0262 | 97.61 | 92.96 | 46.75 | 50569706(96.31) |

| **Supplementary Table 2**. Primers used in this study. | | |
| --- | --- | --- |
| **Gene ID** | **Forward primer** | **Reverse primer** |
| Randomly selected genes | | |
| Bhi01G000575 | AGATCCGCTAGCTCGTCTCA | TCACCATAAGGAGCCCAAGC |
| Bhi04G000584 | GACAGTGGTGACCAGGGATG | TGGACCTTCCAACGCTCTTC |
| Bhi11G001601 | TCAATTCACCGCCAAGCTCT | TCGTGCGTTGTCTCTCACAA |
| Bhi03G002023 | CCTCCGACCTTCTCCACAAC | TGTCGTCATCTACCGGCAAC |
| Bhi11G001731 | AAACAAAAGGGCGCGGGATA | ATGCTCGAATTCCATCCGCA |
| Bhi06G000622 | TGGATGATGATGCTCCGTCC | CGAATCGGAAATGGCAGTCAC |
| Bhi02G000259 | AGAATCTCTCTCCCGGTGCT | CCCCCAAGCAAAGACTGTGA |
| Bhi05G000832 | ACAGGAAAGCAGCCCAATGA | GACTGCATCCTCCACCTGAG |
| Bhi07G001503 | TGAGACAGACGCAAACCGAA | TCCAGTTAGTGTGCAAGCCA |
| TF genes | | |
| Bhi04G000283 | CCATTGCTCGTTTGCTTCCC | TCTCAGGGGCAATTTCGTCC |
| Bhi04G002104 | CGGAGATACGAGTTCCAGGC | GGAAGTTCAGGTGGTCCAGG |
| Bhi07G001177 | TGCAGCAAGGTCACTGGAAA | CCTTGGATTCACGGTAGCGA |
| Bhi08G001625 | CACAAAACCGAGCCATTGGG | TGAATGCCAGAGCTGTCCTC |
| Bhi09G000364 | AATGCTTGCATTGTCAGGCG | TATGAACGTCGGACTCGCTG |
| Bhi12G000380 | GTGCCGCTGCTAAGAATTGG | GTATCTCCTTCCTGCTGGCG |
| Bhi12G001034 | GGCTGCCAAAGATGGCTTTC | TCTCGACGGGGATATCGTCA |
| Bhi12G002174 | AGAAAGTGCTCGGAAACGGT | TGAAATGGGCTGAGGGGATG |
| Bhi08G001865 | TTCCACCACCCTCAATTCGG | GGCGATCGAAAATCACACCG |
| Bhi09G002830 | CTTCTTCGACGCGTTTGCTC | GCCGAGATTTTCGCGTAGTG |
| Bhi11G001816 | CCCATCTCCAGGGAAAGAGC | GCAGAGAAGAGTGGCACAGA |
| Actins | | |
| Bhi10G001911 | ATGTTCACAACCACTGCCGA | GTCGAGCGCAACATAAGCAA |
| Bhi10G000469 | CCAAGTCCGCAGTCAAG | ACCGTCTATCAAGCACCC |


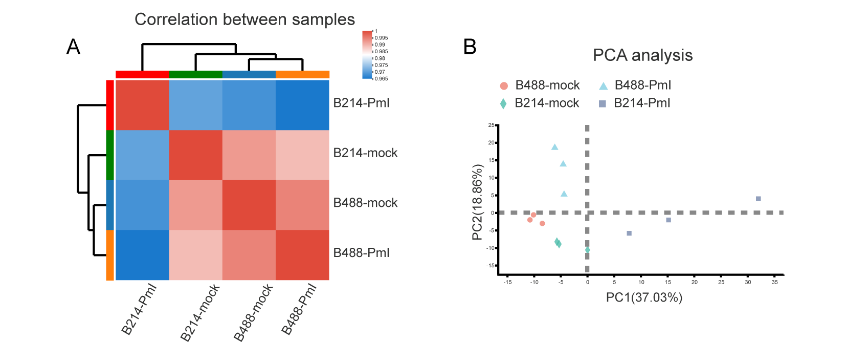


**Supplementary Figure 1**. Quality Evaluation

(A) Correlation analysis. (B) Principal component analysis (PCA) analysis. PCA was performed on the biological replicates of each sample set.


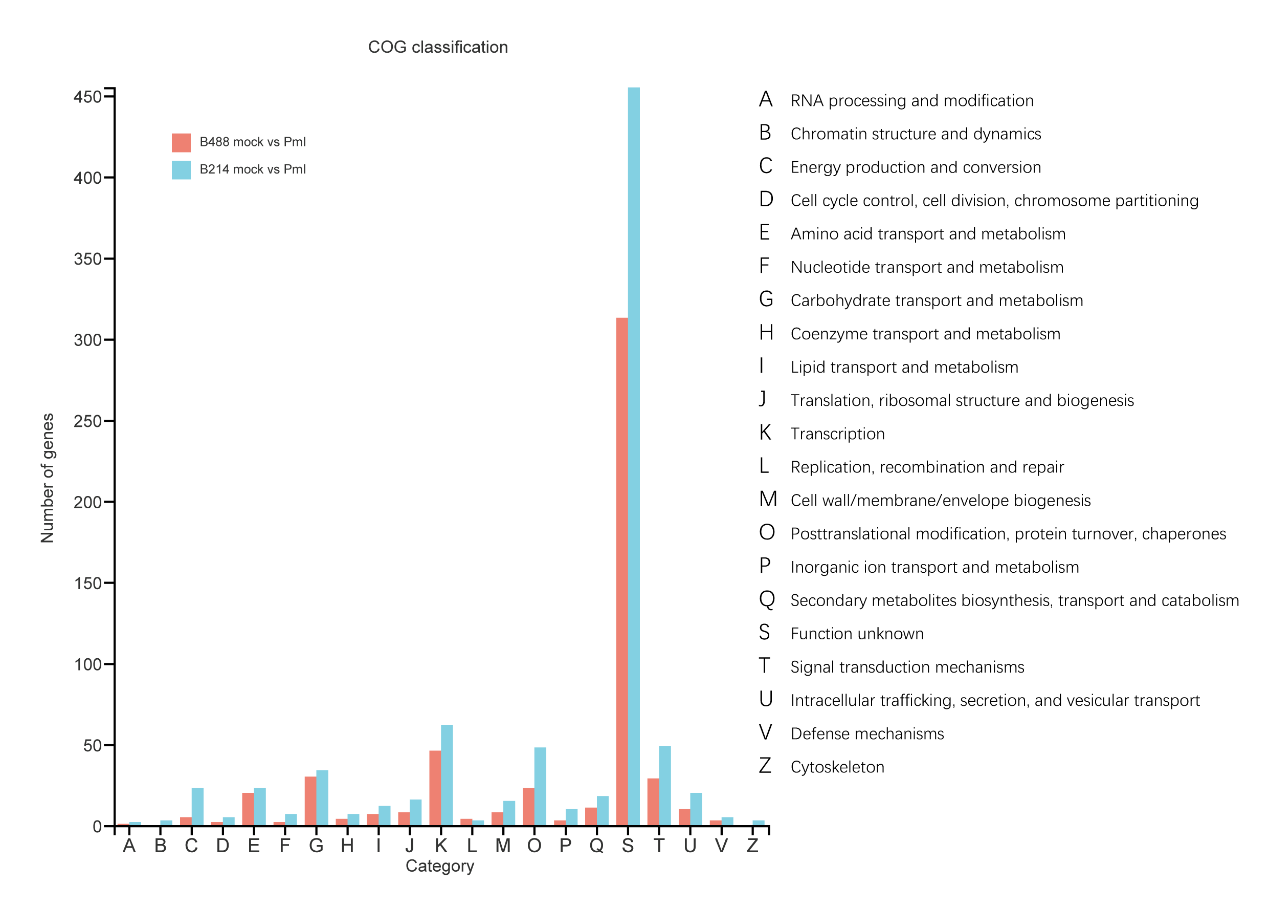


**Supplementary Figure 2**. Clusters of orthologous groups (COG) classification of DEGs.


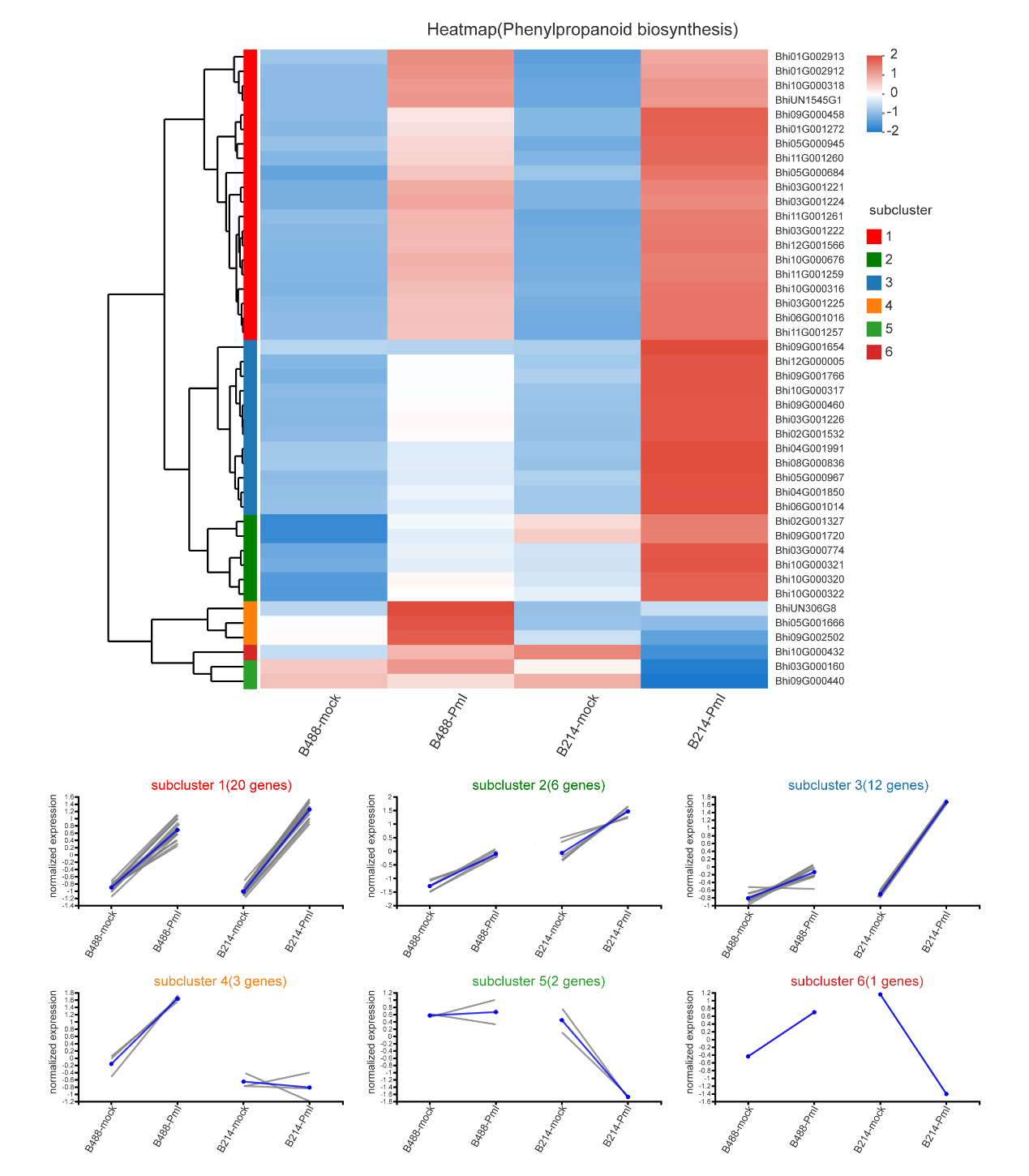


**Supplementary Figure 3**. Cluster analysis of DEGs in Phenylpropanoid biosynthesis pathway.


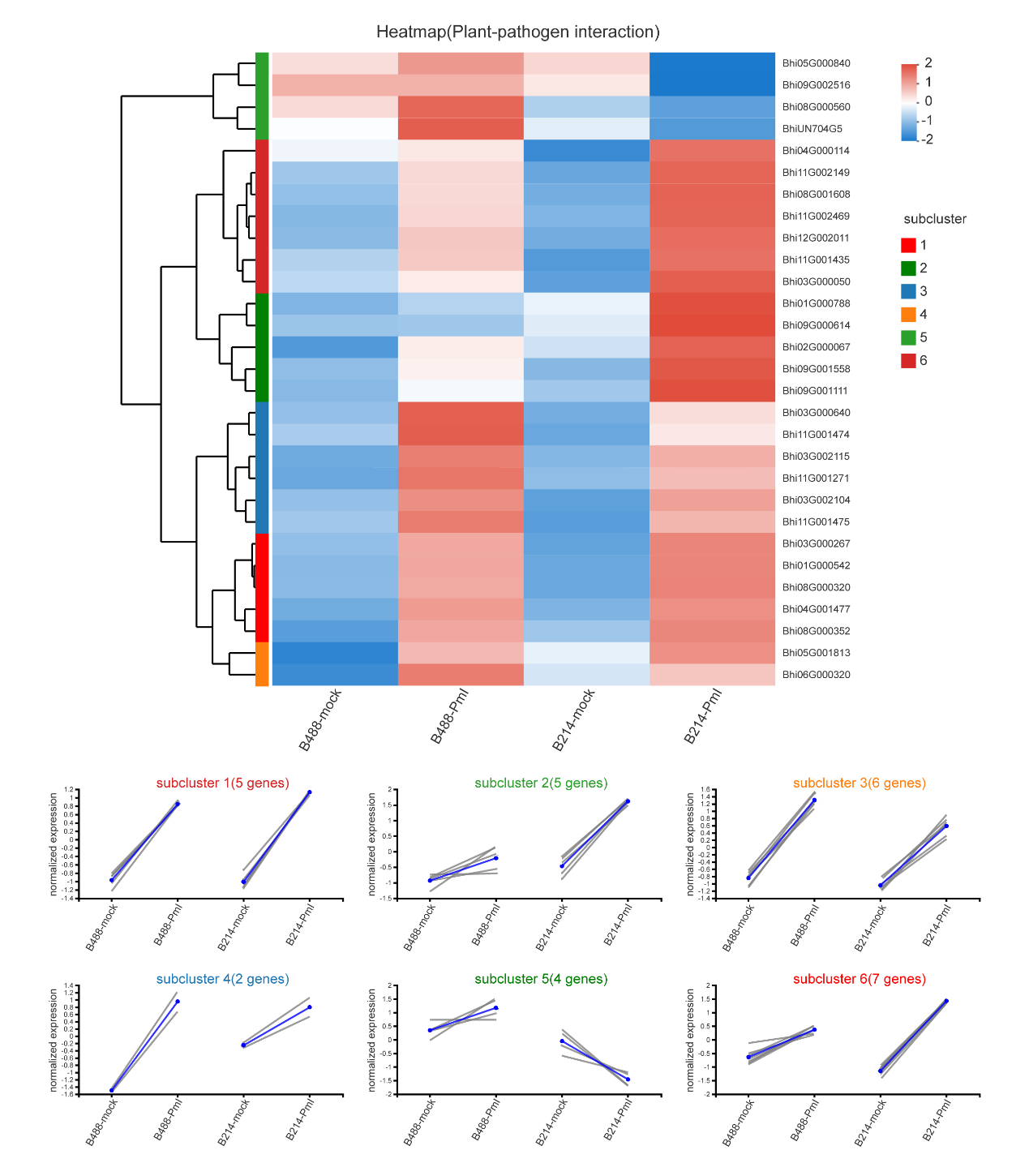


**Supplementary Figure 4**. Cluster analysis of DEGs in Plant-pathogen interaction pathway.


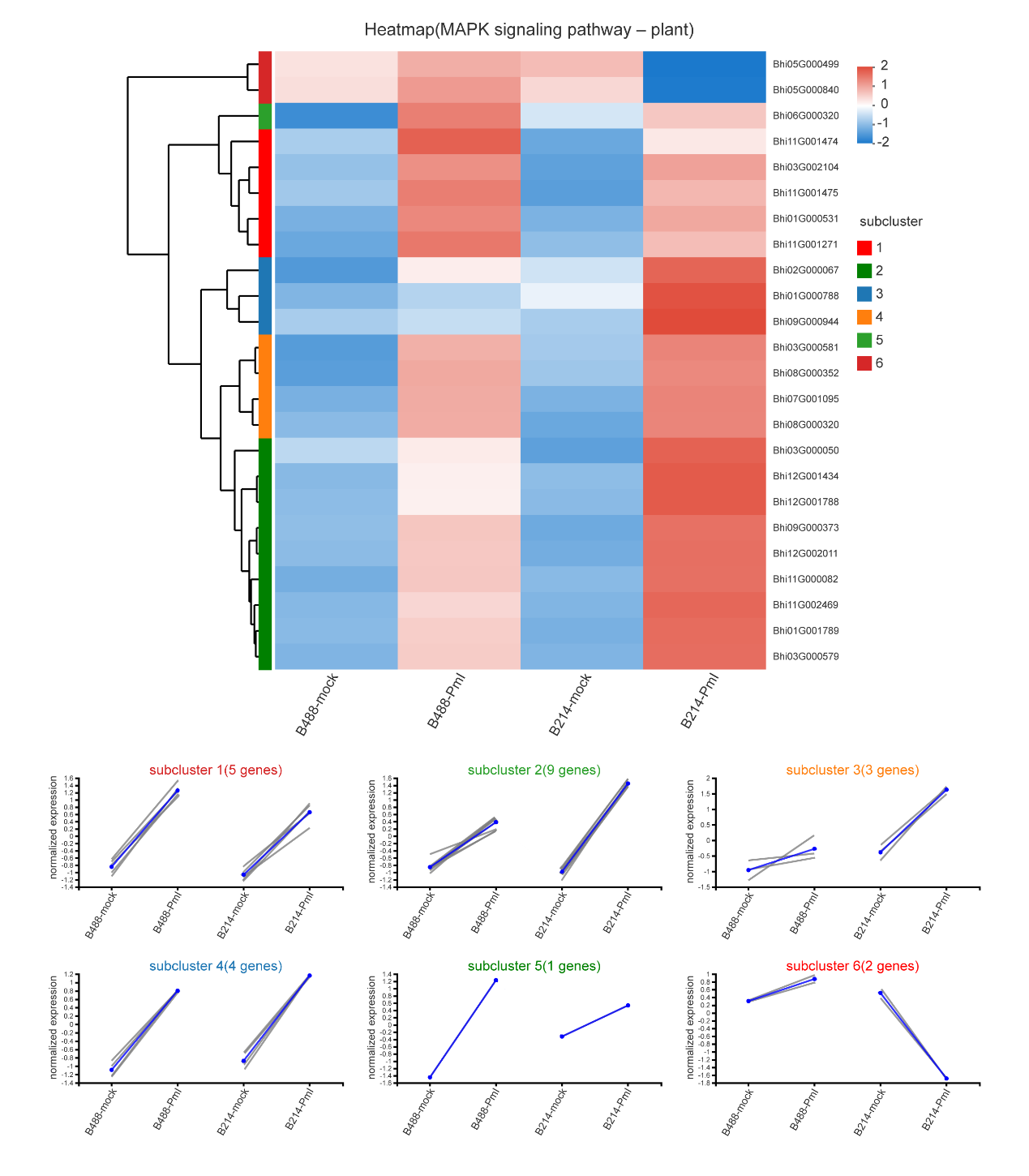


**Supplementary Figure 5**. Cluster analysis of DEGs in MAPK signaling pathway-plant.


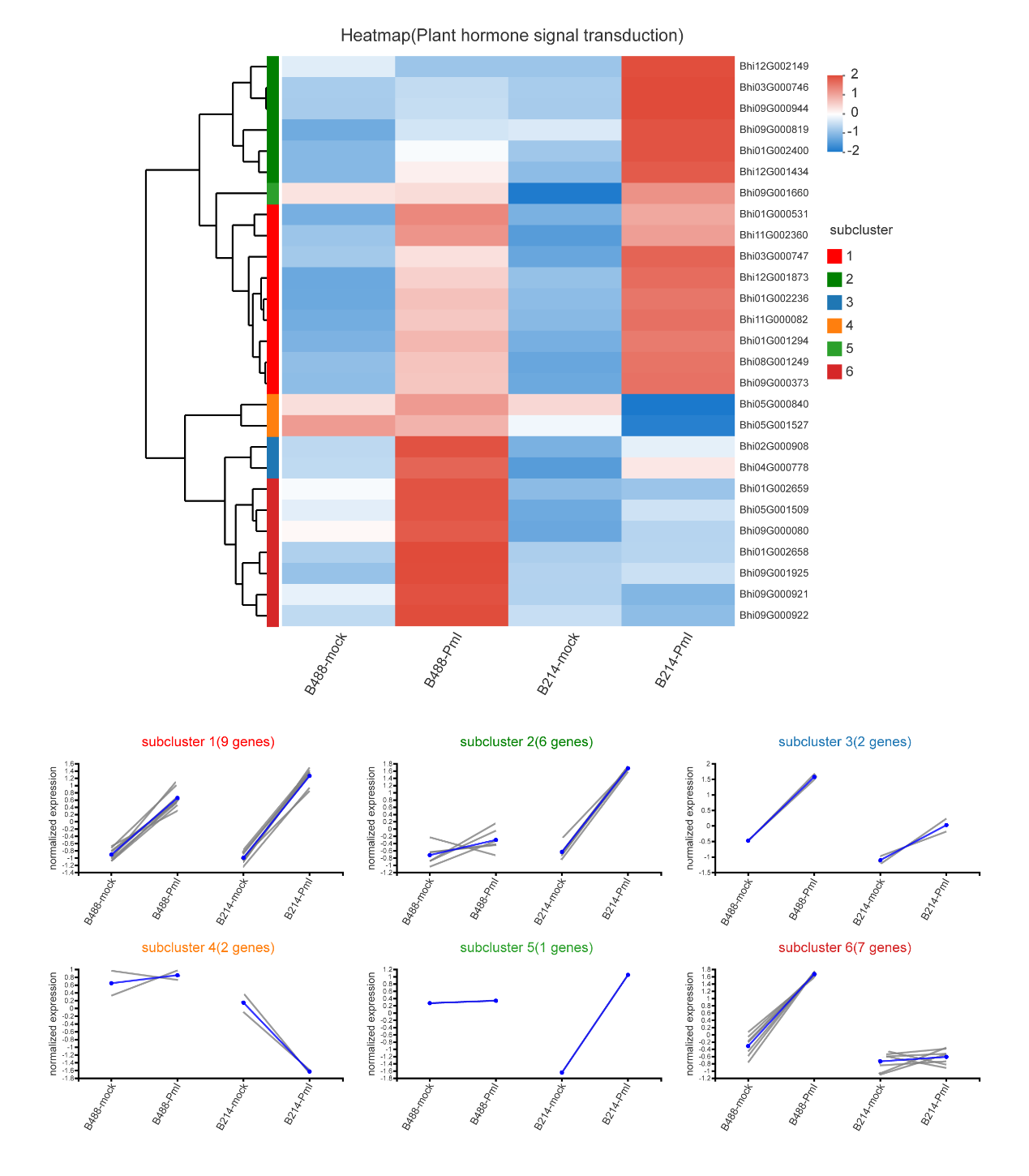


**Supplementary Figure 6**. Cluster analysis of DEGs in plant hormone signal transduction pathway.


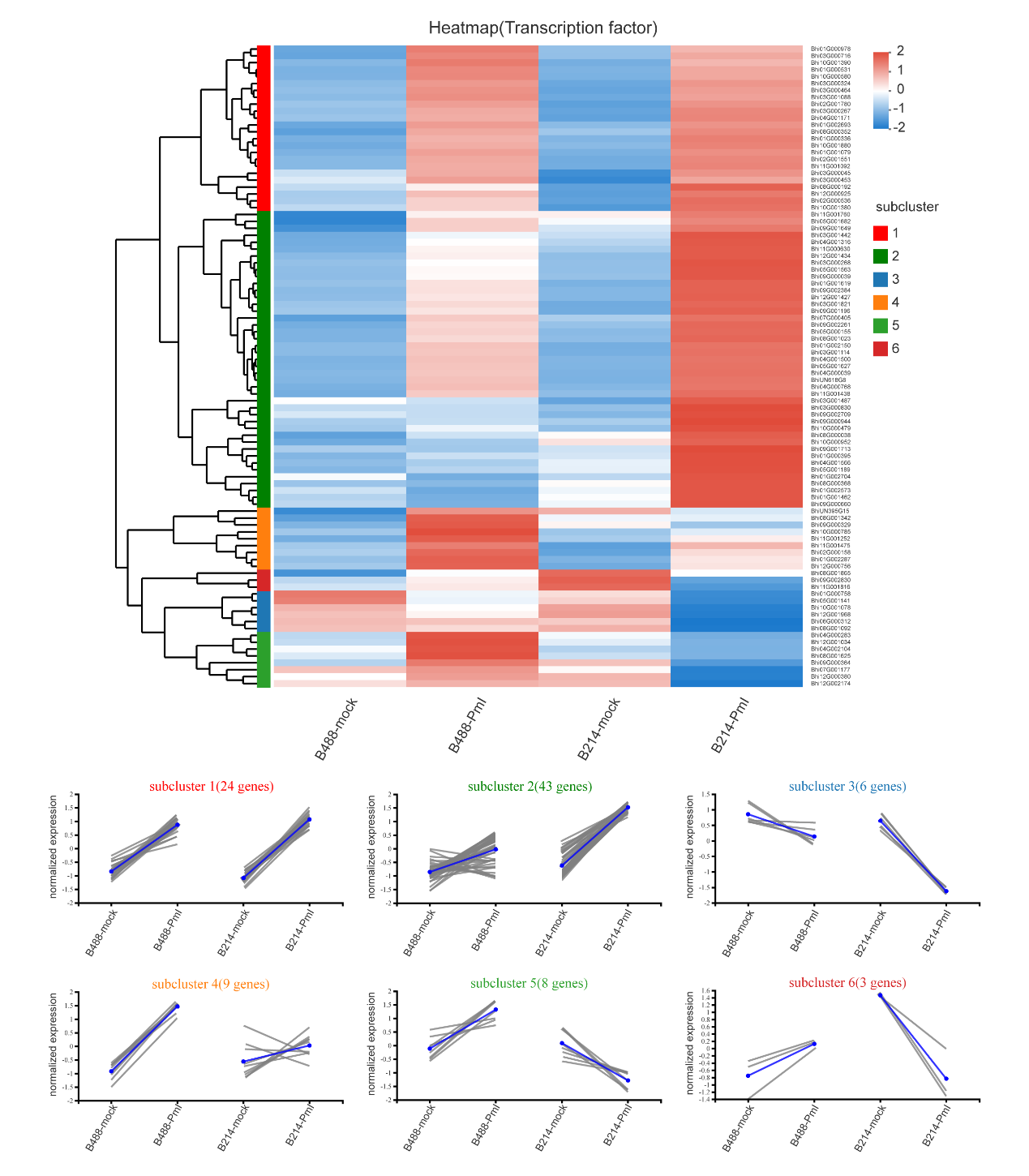


**Supplementary Figure 7**. Cluster analysis of TF genes.
